# Prognostic prediction and treatment options for gastric signet ring cell carcinoma: a SEER database analysis

**DOI:** 10.3389/fonc.2024.1473798

**Published:** 2024-10-21

**Authors:** Chengqing Yu, Jian Yang, Haoran Li, Jie Wang, Kanghui Jin, Yifan Li, Zixiang Zhang, Jian Zhou, Yuchen Tang

**Affiliations:** ^1^ Department of General Surgery, The First Affiliated Hospital of Soochow University, Suzhou, China; ^2^ State Key Laboratory of Radiation Medicine and Protection, Soochow University, Suzhou, China; ^3^ Department of Gastrointestinal Surgery, Affiliated Changzhou No.2 People’s Hospital of Nanjing Medical University, Changzhou Medical Center, Changzhou, China

**Keywords:** gastric cancer, signet ring cell carcinoma, SEER database, nomogram, survival

## Abstract

**Background:**

In recent years, the overall incidence of gastric cancer has decreased. However, the incidence of gastric signet ring cell carcinoma (SRCC) is still increasing year by year. Compared with other subtypes (non-SRCC) such as adenocarcinoma, SRCC usually exhibits a more aggressive biological behavior. Therefore, studying the prognostic differences and factors associated with SRCC is essential to improve the accuracy of diagnosis and prognosis. The purpose of this study was to investigate the prognostic factors influencing the prognosis of patients with SRCC and to develop personalized treatments for different subgroups of patients.

**Methods:**

The data on gastric SRCC patients and gastric adenocarcinoma (AC) patients from 1992 to 2020 was obtained from the Surveillance, Epidemiology, and End Results (SEER) database. The data of gastric SRCC as the external validation group was reviewed from the First Affiliated Hospital of Soochow University. The overall survival (OS) and cancer specific survival (CSS) at 1 and 2 years were predicted for SRCC patients by constructing prognostic nomograms. A series of validation methods, including Akaike information criterion (AIC), decision curve analysis (DCA), calibration curve analysis, the concordance index (C-index) and the area under the receiver operating characteristic (AUC) curve, were used to verify the accuracy and reliability of the models.

**Results:**

In all, 549 patients with SRCC were included after propensity score matching (PSM). Multivariate Cox regression analysis showed that T stage, N stage, M stage and surgical approach were independent risk factors affecting the prognosis of SRCC patients. A prognostic nomogram was constructed and validated as an accurate model for SRCC patients after scoring by receiver operating characteristic curve (ROC) curves and calibration plots. The patients were further divided into high-risk and low-risk groups, and the Kaplan-Meier curves showed that SRCC patients in the low-risk group could receive only surgery without chemotherapy, while chemotherapy plus surgery was a better option for SRCC patients in the high-risk group.

**Conclusion:**

The prognosis for SRCC was less favorable than that of AC in terms of CSS. The nomograms were developed and validated to predict OS and CSS in patients with SRCC, helping in developing appropriate individualized treatment schedules.

## Introduction

According to the latest global cancer burden data (Globocan 2020), the number of new cases of gastric cancer worldwide is 1,089,000, which ranks fifth among all malignancies, while the number of deaths is 768,000, which ranks third (1). With advances in the standard treatment of *Helicobacter pylori* (HP) infection, the overall incidence of gastric cancer has decreased (2). However, the incidence of gastric signet ring cell carcinoma (SRCC) continues to increase every year (3). SRCC is a histological subtype of gastric cancer defined by the presence of mucinous cells, which account for more than 50% of the tumor volume and extrude into the cellular ridges. This subtype is generally considered to have a poor prognosis (4). Clinically, SRCC is most prevalent in young women and has a high incidence of distant metastasis, which results in an unfavorable prognosis. SRCC typically demonstrates more aggressive biological behavior than other subtypes (non-SRCC) such as adenocarcinoma (5, 6). The prognosis of SRCC remains controversial in the literature. Several studies have indicated that patients diagnosed with SRCC tend to have a relatively favorable prognosis in the early stages but a poorer prognosis in the late stages compared with patients with other histological subtypes (7–9). However, several Western studies have analyzed SRCC versus non-SRCC, but the preliminary conclusions are not consistent with those of Eastern countries (10, 11). A literature review revealed that several factors, including age, tumor size, tumor-node-metastasis (TNM) stage, epidermal growth factor receptor (EGFR) status, surgical approach, radiation therapy, and chemotherapy, can influence the prognosis of SRCC patients (11–18). Therefore, it is crucial to investigate the prognostic differences and influencing factors associated with SRCC to enhance diagnostic and prognostic accuracy.

The poor prognosis of SRCC patients is attributable to a low rate of curative resection and a poor response to radiotherapy. Furthermore, the low expression of human epidermal growth factor receptor-2 (HER-2) in these patients limits the efficacy of targeted therapy for the treatment of SRCC (19, 20). The National Comprehensive Cancer Network (NCCN) and the American Society of Clinical Oncology (ASCO) do not recommend any specific treatment for SRCC in their respective clinical practice guidelines. Consequently, the development of a unique multimodal treatment regimen for SRCC is urgently needed. In clinical practice, the American Joint Committee on Cancer (AJCC) staging system has a significant impact on prognosis and serves as a key tool for clinicians in developing and implementing treatment strategies. In an era characterized by an abundance of data, data mining techniques offer an opportunity to extract potentially valuable insights from diverse sources of information. Nomograms have become common tools for assessing the prognosis of cancer patients and for personal prediction of patient survival. Nomograms have been shown to be a more appropriate clinical tool for patient management than the AJCC staging system. Prior nomograms for SRCC have made significant explorations of prognostic factors; nevertheless, either the treatment data have not been fully incorporated or the nomograms have not been fully used for the purpose of determining risk and guiding treatment strategies, thereby limiting the clinical utility of these nomograms (16, 21–30).

In this study, a nomogram was established and validated for overall survival (OS) and cancer specific survival (CSS) in SRCC patients based on important prognostic factors with publicly available data from the Surveillance, Epidemiology, and End Results (SEER) database. In addition, the concordance index (C-index), calibration plots, and decision curve analysis (DCA) were used to assess the discriminatory power and clinical utility of the nomogram. Furthermore, the nomogram was used to stratify risk and was combined with clinical treatment to predict survival and optimize the clinical management of SRCC patients.

## Materials and methods

### Patient selection

The SEER database is a publicly available cancer reporting system sponsored by The Surveillance Research Program of the Division of Cancer Control and Population Sciences, National Cancer Institute Information Management Services, Inc. The SEER database provides clinical information about cancer patients, such as age, gender, race, primary tumor site, tumor size, tumor grade, tumor stage, pathology type, survival time, cause of death, and chemotherapy and radiotherapy status. SEER*Stat software (version 8.4.3, SEER Research Data, 12 Registries, Nov 2022 Sub (1992-2020) database) was used for this study (https://seer.cancer.gov/seerstat/).

Patients with gastric cancer with a primary site in the stomach according to the International Classification of Diseases of Oncology (ICD-O) with a pathology type code of 8140/3: adenocarcinoma, 8141/3: scirrhous adenocarcinoma, 8142/3: linitis plastica, 8143/3: superficial spreading adenocarcinoma, 8144/3: adenocarcinoma, intestinal type, 8262/3: villous adenocarcinoma, 8323/3: mixed cell adenocarcinoma, and 8490/3: signet ring cell carcinoma were included in this study. In all, 83,258 patients with gastric cancer were included. To reduce noise, we eliminated patients with unknown survival time (6196 patients), unknown stage (50,380 patients), uncertain T stage, including Tis, Tx, and T0 (51,162 patients), uncertain N stage, including NX (51,450 patients), and unknown M stage (49,533 patients). Ultimately, 3,094 patients were enrolled in this study, including 2,535 patients with adenocarcinoma (AC) and 559 with SRCC.

### Follow-up of patients

The patients with gastric SRCC in the First Affiliated Hospital of Soochow University were followed up through telephone. Research involving human subjects was approved by the Ethics Committee of the First Affiliated Hospital of Soochow University [(2024) Len Research Grant No. 347]. The start date of the follow-up was the first day after surgery and ended on June 30, 2024. The follow-up index were Overall survival (OS) and Cancer-specific survival (CSS), and the outcome event was death.

### Clinical variables

Patient variables, such as age, race, gender, tumor size, tumor site, TNM stage, number of lymph nodes, number of positive lymph nodes, treatment (surgery, radiotherapy, and chemotherapy), survival time, status and cause of death, were collected from the SEER database. Overall survival (OS) was the primary endpoint and was determined from the date of diagnosis until the date of death from any cause or the last follow-up. Cancer-specific survival (CSS) was the secondary endpoint and was determined from the date of diagnosis until the date of death from cancer or the last follow-up visit. In the SEER cohort, CSS was defined by the SEER cause-specific death category. Patients were categorized into three age groups according to the World Health Organization (WHO) criteria: young (<45 years), middle-aged (45-65 years) and old (>65 years).

### Establishment and validation of nomograms

In this study, to determine the prognostic factors for SRCC patients, proportional risk hypothesis testing was performed using the “survival” package in R. Six clinical characteristics were initially screened by univariate Cox regression analysis, and samples for which *p <*0.05 were included in the multivariate Cox regression analysis. Then, 1-year and 2-year nomograms were constructed based on the independent prognostic risk factors derived from the multivariate Cox regression analysis using the “regplot” package. Prognostic nomograms based on the results of the multivariate analysis were assessed for sensitivity, specificity, discrimination, and calibration. Sensitivity and specificity were determined by receiver operating characteristic (ROC) curve analysis using the “timeROC” package, and the area under the curve (AUC) was determined. Differentiation was quantified by calculating the C-index using the “rms” package. Calibration was assessed based on calibration curves, which were analyzed using the “rms” package and then plotted using the “ggplot2” package to analyze the correlation between predicted probabilities and actual results. DCA was performed by fitting the prognostic model using the “survival” package, and DCA analysis was performed with the “stdca” package. Furthermore, the bootstrap method was used to validate the stability and accuracy of the model by replicating the sample 1000 times and calculating the C-index, Akaike information criterion (AIC), and AUC.

### Survival subgroup analysis of the nomogram

A prognostic risk score nomogram was constructed. Using the median risk score as the cutoff point, patients were divided into high-risk and low-risk groups, and survival analyses were performed using the “survival” package to assess the difference in survival between the AC group and the SRCC group, as well as the performance of different treatment modalities for SRCC patients.

### Statistical analysis

In the present study, categorical variables were compared using Pearson’s chi-square test or Yates correction, while continuous variables were compared using Fisher’s test or Wilcoxon’s test. To balance the baselines of the AC group and the SRCC group, propensity score matching (PSM) was performed using logistic regression with a ratio of 1:1 and a caliper width of 0.01 without replacement.

Statistical analyses were conducted using R software (R Project for Statistical Computing, RRID: SCR_001905) version 4.3.2. Statistical significance was defined as *p <*0.05.

## Results

### Characteristics of patients with AC and SRCC

**Figure 1:**
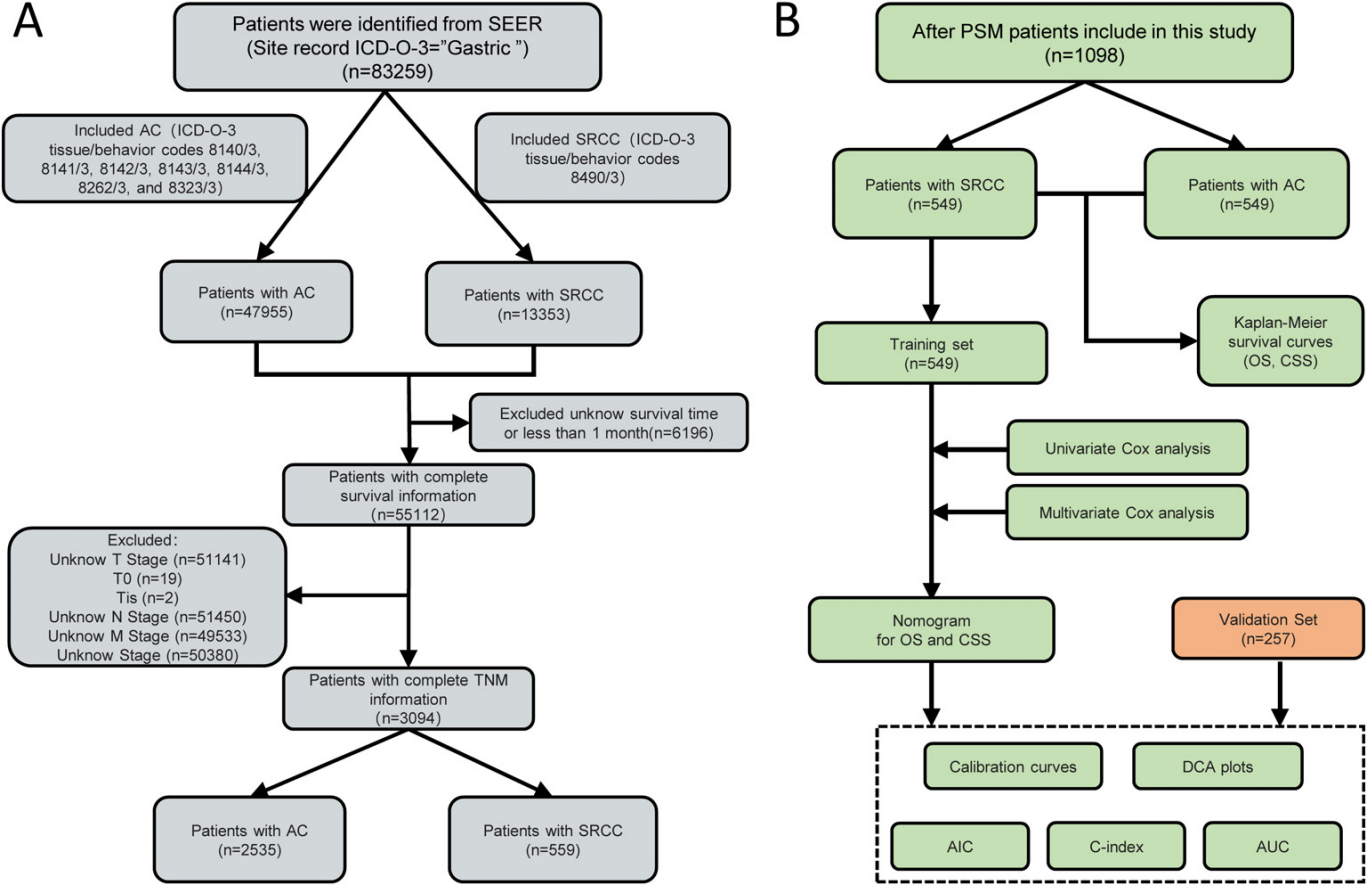
A flow chart of the study. **(A)** Flow chart for screening patients. **(B)** Flow chart for constructing the prognostic model. of signet ring cell carcinoma (SRCC) patients. AC, Adenocarcinoma; SRCC, Signet ring cell carcinoma; PSM, Propensity score matching; TNM, tumor-node-metastasis; SEER, Surveillance, epidemiology and end results; OS, Overall survival; CSS, Cancer Specific Survival; AIC, Akaike information criterion; C-index, concordance index; AUC, Area Under the Curve; DCA, Decision Curve Analysis.

Based on the inclusion and exclusion criteria, 3094 patients (2535 with AC and 559 with SRCC) from the SEER database who were diagnosed between 1992 and 2020 were enrolled in this study. Meanwhile, a total of 257 SRCC patients were collected from our hospital (**Figure 1**). Several baseline clinical characteristics were significantly different between the AC and SRCC groups (*p* <0.001; **Table 1**). Compared with AC patients, SRCC patients were more likely to be female (56.2% vs. 32.0%, *p* <0.001) and aged <45 years (13.8% vs. 4.5%, *p* = 0.018). Regarding tumor characteristics, more patients in the SRCC group had stage IV, T4, or N4 disease (*p* <0.001). Moreover, significant differences were also observed in the incidence of distant metastasis between the two groups (25.2% vs. 15.9%, *p* <0.001). Interestingly, we found that the percentages of AC and SRCC patients who did not undergo surgery were similar (33.5% vs. 34.0%, *p* >0.05), but the proportion of patients who underwent total gastrectomy for SRCC was obviously greater than that for AC patients (13.6% vs. 6.0%, *p* <0.001).

**Table 1:** Baseline characteristics of AC and SRCC before and after propensity score matching.

Subject	Before PSM			After PSM		
Characteristics	AC	SRCC	*P-* value	AC	SRCC	*P-* value
n	2535	559		549	549	
Age, n (%)			< 0.001			0.692
<45 years	113 (4.5%)	77 (13.8%)		61 (11.1%)	70 (12.8%)	
45-65 years	754 (29.7%)	221 (39.5%)		225 (41.0%)	218 (39.7%)	
>65 years	1668 (65.8%)	261 (46.7%)		263 (47.9%)	261 (47.5%)	
Race, n (%)			0.276			0.060
White	1650 (65.1%)	353 (63.1%)		66 (12.0%)	43 (7.8%)	
Black	234 (9.2%)	43 (7.7%)		164 (29.9%)	154 (28.1%)	
Other (American Indian/AK Native, Asian/Pacific Islander)	630 (24.9%)	159 (28.4%)		313 (57.0%)	348 (63.4%)	
Unknown	21 (0.8%)	4 (0.7%)		6 (1.1%)	4 (0.7%)	
Gender, n (%)			< 0.001			0.952
Female	810 (32.0%)	314 (56.2%)		303 (55.2%)	304 (55.4%)	
Male	1725 (68.0%)	245 (43.8%)		246 (44.8%)	245 (44.6%)	
Primary Site, n (%)			< 0.001			< 0.001
Cardia	1094 (43.2%)	42 (7.5%)		85 (15.5%)	42 (7.7%)	
Fundus of stomach	77 (3.0%)	18 (3.2%)		22 (4.0%)	17 (3.1%)	
Body of stomach	232 (9.2%)	97 (17.4%)		59 (10.7%)	97 (17.7%)	
Gastric antrum	459 (18.1%)	150 (26.8%)		112 (20.4%)	149 (27.1%)	
Pylorus	89 (3.5%)	30 (5.4%)		23 (4.2%)	30 (5.5%)	
Lesser curvature of stomach	214 (8.4%)	47 (8.4%)		56 (10.2%)	47 (8.6%)	
Greater curvature of stomach	72 (2.8%)	17 (3.0%)		32 (5.8%)	16 (2.9%)	
Overlapping lesion of stomach	134 (5.3%)	67 (12.0%)		72 (13.1%)	65 (11.8%)	
Stomach	164 (6.5%)	91 (16.3%)		88 (16.0%)	86 (15.7%)	
T, n (%)			< 0.001			0.279
T1	948 (37.4%)	185 (33.1%)		180 (32.8%)	183 (33.3%)	
T2	337 (13.3%)	68 (12.2%)		73 (13.3%)	68 (12.4%)	
T3	872 (34.4%)	155 (27.7%)		175 (31.9%)	153 (27.9%)	
T4	378 (14.9%)	151 (27.0%)		121 (22.0%)	145 (26.4%)	
N, n (%)			< 0.001			0.026
N0	1506 (59.4%)	346 (61.9%)		330 (60.1%)	338 (61.6%)	
N1	604 (23.8%)	64 (11.4%)		97 (17.7%)	64 (11.7%)	
N2	225 (8.9%)	56 (10.0%)		49 (8.9%)	56 (10.2%)	
N3	200 (7.9%)	93 (16.6%)		73 (13.3%)	91 (16.6%)	
M, n (%)			< 0.001			0.532
M0	2131 (84.1%)	418 (74.8%)		406 (74.0%)	415 (75.6%)	
M1	404 (15.9%)	141 (25.2%)		143 (26.0%)	134 (24.4%)	
Stage Group, n (%)			< 0.001			0.522
I	908 (35.8%)	174 (31.1%)		173 (31.5%)	172 (31.3%)	
II	624 (24.6%)	123 (22.0%)		129 (23.5%)	122 (22.2%)	
III	564 (22.2%)	121 (21.6%)		102 (18.6%)	121 (22.0%)	
IV	439 (17.3%)	141 (25.2%)		145 (26.4%)	134 (24.4%)	
Surg Prim Site, n (%)			< 0.001			0.122
Local tumor excision	198 (7.8%)	12 (2.1%)		191 (34.8%)	186 (33.9%)	
No surgery	848 (33.5%)	190 (34.0%)		18 (3.3%)	11 (2.0%)	
Partial gastrectomy	1336 (52.7%)	280 (50.1%)		288 (52.5%)	277 (50.5%)	
Total gastrectomy	153 (6.0%)	76 (13.6%)		52 (9.5%)	74 (13.5%)	
Unknown	0 (0%)	1 (0.2%)		0 (0%)	1 (0.2%)	
Surg/Rad Seq, n (%)			< 0.001			0.584
No radiation	2063 (81.4%)	511 (91.4%)		506 (92.3%)	501 (91.3%)	
Radiation	472 (18.6%)	48 (8.6%)		43 (7.8%)	48 (8.7%)	
Chemotherapy, n (%)			0.133			0.654
No/Unknown	915 (36.1%)	183 (32.7%)		186 (33.9%)	179 (32.6%)	
Yes	1620 (63.9%)	376 (67.3%)		363 (66.1%)	370 (67.4%)	
Tumor Size, median (IQR)	34 (20, 50)	40 (17, 60)	0.310	55 (30, 999)	70 (30, 999)	0.143
Regional nodes examined, median (IQR)	10 (0, 23)	15 (0, 27)	< 0.001	15 (0, 27)	15 (0, 27)	0.653
Regional nodes positive, median (IQR)	0 (0, 3)	1 (0, 7)	< 0.001	7 (0, 98)	8 (0, 98)	0.614

AC, Adenocarcinoma; SRCC, Signet ring cell carcinoma; PSM, propensity score matching; T, tumor; N, node; M, metastasis.

### Survival analysis and PSM

**Figure 2:**
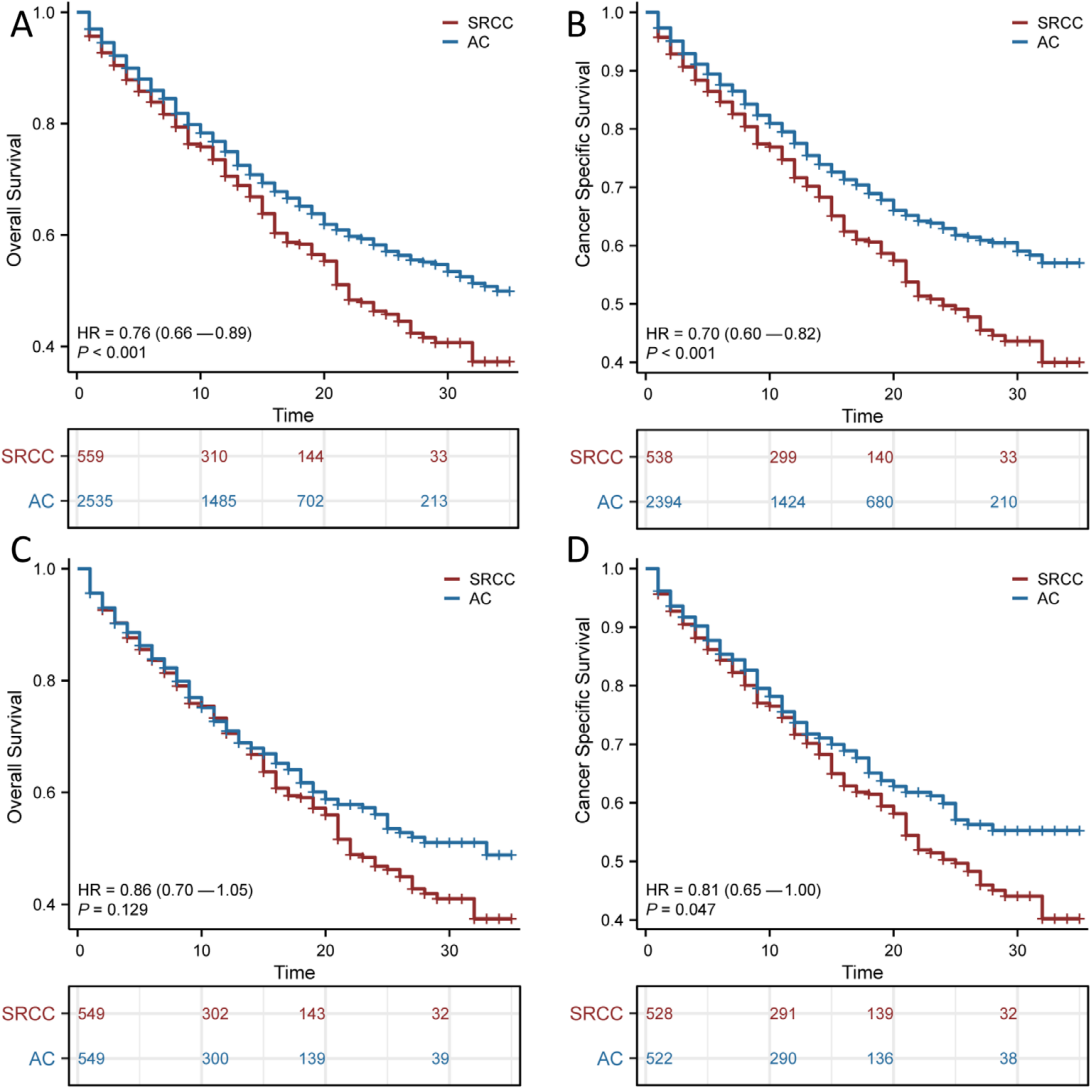
Survival outcomes before and after propensity score matching (PSM). Overall survival (OS) **(A)** and Cancer Specific Survival (CSS) **(B)** in patients with adenocarcinoma (AC) and signet ring cell carcinoma (SRCC) before PSM; OS **(C)** and CSS **(D)** in patients with AC and SRCC after PSM.

We found that AC patients had a significantly better survival probability than SRCC patients (OS, *p <*0.001; CSS, *p <*0.001; **Figures 2A, B**). However, we observed significant structural differences between the 2 groups. To balance the confounding factors, we performed PSM at a 1:1 ratio. As shown in **Table 1**, we matched 549 patients with AC with 549 patients with SRCC. After PSM, the baseline characteristics (age, race, gender, summary stage, T stage, N stage, chemotherapy and radiation status, primary site surgery, and metastatic sites) were all balanced (*p* > 0.05). The propensity score distribution between AC patients and SRCC patients became similar after PSM, and the difference in clinicopathological characteristics between the 549 matched pairs was significantly decreased, which resulted in good balance across all covariates (**Supplementary Figures 1A, B**). We then performed a KM survival analysis and found that patients with SRCC had poorer CSS than those with AC (*p* =0.047; **Figure 2D**). However, the OS rates of SRCC and AC patients after PSM were inconsistent with these results. After PSM, the median OS times were 22 and 33 months for the SRCC and AC groups, respectively, but this difference was not significant (*p* = 0.129; **Figure 2C**). The results were similar, even after a subgroup analysis was performed according to cancer stage (**Supplementary Figure 2**).

**Figure 3:**
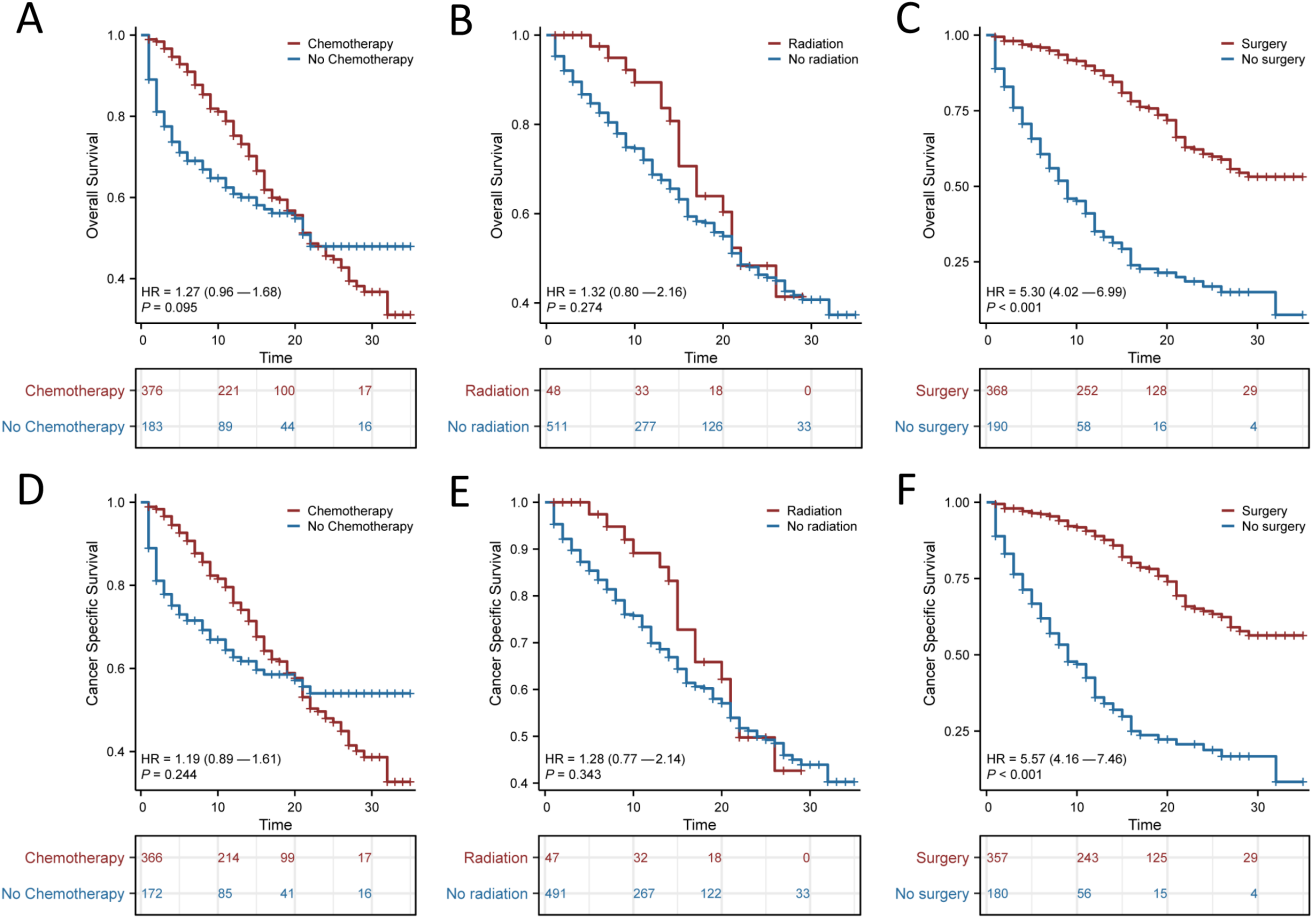
Kaplan-Meier survival curves for signet ring cell carcinoma (SRCC) patients receiving different treatment modalities after propensity score matching (PSM). Overall survival (OS) **(A)** and Cancer Specific Survival (CSS) **(D)** for patients with or without chemotherapy; OS **(B)** and CSS **(E)** for patients with or without radiotherapy; OS **(C)** and CSS **(F)** for patients with or without surgery.

The use of surgery, chemotherapy and radiotherapy has increased as the mainstays of treatment for SRCC. We next explored the effects of these variables on patient OS and CSS, and the results suggested that surgery had a significant impact on patient prognosis (*p* < 0.001; **Figure 3**). The median OS was 15 months (95% CI, 7.1–22.9 months) for those who underwent surgery and 9 months (95% CI, 7–11 months) for those who did not. The median CSS for those who underwent surgery was 11 months (95% CI, 2 months–20 months), and the median CSS for those who did not undergo surgery was 9 months (95% CI, 7 months–12 months). In contrast, chemotherapy alone or radiotherapy alone did not significantly improve the OS or CSS of patients with SRCC.

### Univariate and multivariate analyses

Univariate and multivariate Cox regression analyses were performed to determine the potential clinical characteristics that may influence the prognosis of SRCC patients. No surgery (*p* < 0.001), T4 stage (*p <*0.001), N2 stage (*p* =0.032), N3 stage (*p* =0.001) and metastasis (*p* = 0.002) were identified as independent risk factors for decreased OS in patients with SRCC (**Table 2**). Moreover, no surgery (*p* < 0.001), T4 stage (*p* =0.003), N2 stage (*p* =0.006), N3 stage (*p* < 0.001) and metastasis (*p* = 0.007) were determined to be independent protective factors associated with the CSS of SRCC patients (**Supplementary Table 1**).

**Table 2:** Univariate and multivariate analyses of the SRCC patients for overall survival.

Characteristics	Total(N)	Univariate analysis		Multivariate analysis	
		Hazard ratio (95% CI)	*P*- value	Hazard ratio (95% CI)	*P*- value
Age	340				
<45 years	48	Reference			
45-65 years	133	1.652 (0.851 - 3.205)	0.138		
>65 years	159	1.571 (0.812 - 3.039)	0.180		
Race	340				
Other (American Indian/AK Native, Asian/Pacific Islander)	111	Reference			
White	200	0.848 (0.556 - 1.292)	0.442		
Black	27	1.187 (0.550 - 2.564)	0.662		
Unknown	2	1.630 (0.223 - 11.940)	0.631		
Gender	340				
Female	190	Reference		Reference	
Male	150	1.552 (1.048 - 2.299)	0.028	1.380 (0.911 - 2.092)	0.128
Primary Site	340				
Cardia	24	Reference			
Fundus of stomach	10	0.000 (0.000 - Inf)	0.995		
Body of stomach	57	0.611 (0.267 - 1.400)	0.244		
Gastric antrum	104	0.706 (0.335 - 1.485)	0.358		
Pylorus	22	0.428 (0.143 - 1.281)	0.129		
Lesser curvature of stomach	39	0.508 (0.206 - 1.253)	0.142		
Greater curvature of stomach	10	1.299 (0.434 - 3.887)	0.640		
Overlapping lesion of stomach	29	0.715 (0.283 - 1.803)	0.477		
Stomach	45	0.891 (0.396 - 2.003)	0.780		
Surg Prim Site	340				
Partial gastrectomy	213	Reference		Reference	
No surgery	64	6.446 (4.132 - 10.056)	< 0.001	16.403 (7.827 - 34.376)	< 0.001
Local tumor excision	7	0.000 (0.000 - Inf)	0.993	0.000 (0.000 - Inf)	0.994
Total gastrectomy	56	1.261 (0.707 - 2.250)	0.432	1.490 (0.829 - 2.678)	0.182
T	340				
T1	112	Reference		Reference	
T2	35	0.548 (0.162 - 1.852)	0.333	0.515 (0.149 - 1.777)	0.294
T3	99	2.479 (1.424 - 4.316)	0.001	1.734 (0.956 - 3.145)	0.070
T4	94	3.634 (2.106 - 6.273)	< 0.001	3.215 (1.654 - 6.251)	< 0.001
N	340				
N0	187	Reference		Reference	
N1	44	0.582 (0.263 - 1.286)	0.181	1.492 (0.570 - 3.904)	0.415
N2	40	1.424 (0.819 - 2.478)	0.211	2.414 (1.081 - 5.387)	0.032
N3	69	1.869 (1.175 - 2.974)	0.008	3.391 (1.614 - 7.126)	0.001
M	340				
M0	284	Reference		Reference	
M1	56	6.261 (4.023 - 9.745)	< 0.001	2.221 (1.344 - 3.669)	0.002
Surg/Rad Seq	340				
No	307	Reference			
Yes	33	1.228 (0.671 - 2.245)	0.505		
Chemotherapy	340				
No/Unknown	123	Reference			
Yes	217	1.005 (0.668 - 1.513)	0.980		
Tumor Size Summary	340	1.004 (1.001 - 1.007)	0.007	1.000 (0.995 - 1.005)	0.928

SRCC, Signet ring cell carcinoma; T, tumor; N, node; M, metastasis.

### Development and validation of the nomogram

**Figure 4:**
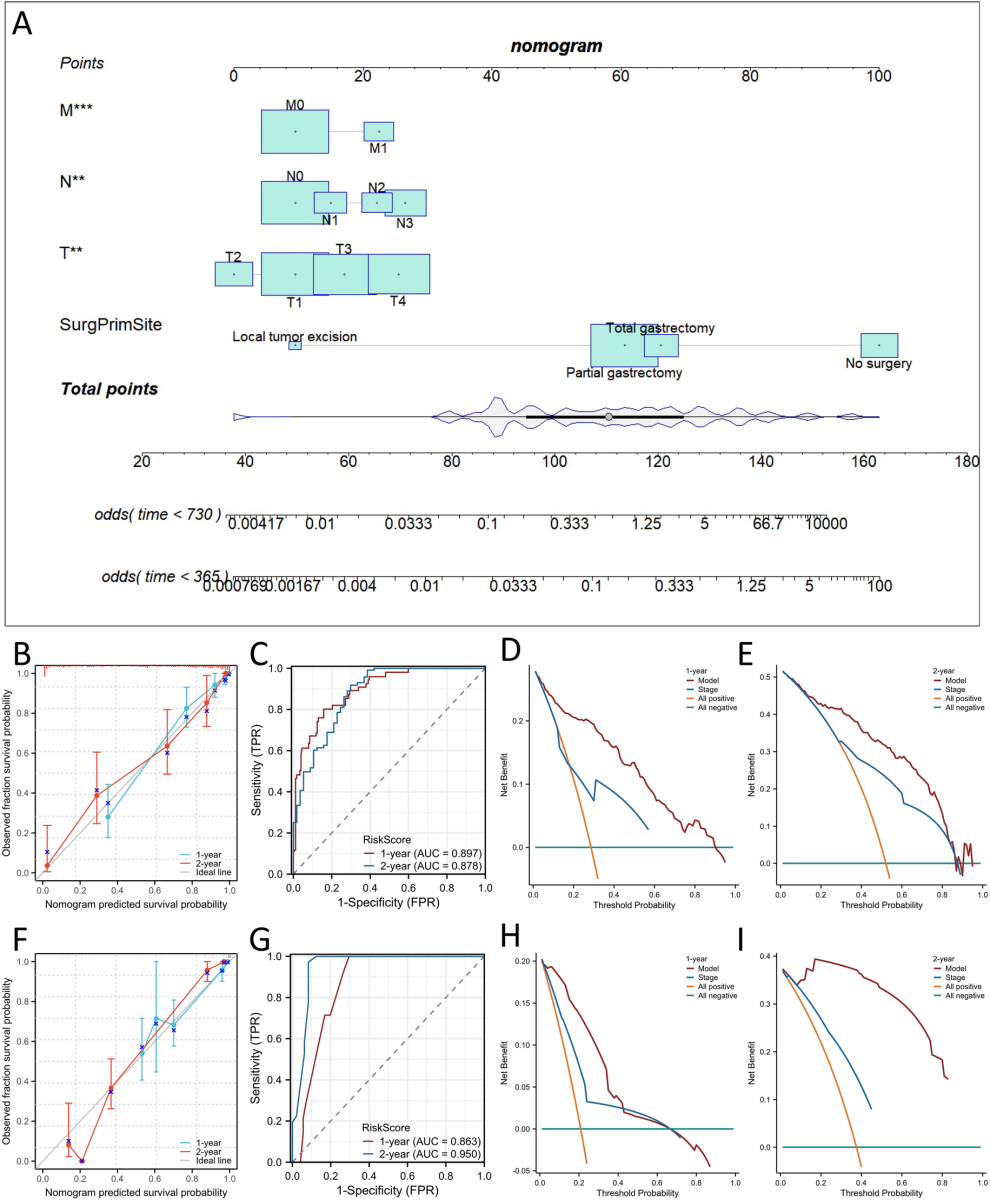
Construction of nomograms for predicting Overall survival (OS) in signet ring cell carcinoma (SRCC) patients and validation of their efficacy and accuracy. Nomograms predicting 1- and 2-year OS **(A)** in patients with SRCC. Calibration plot of the nomogram for predicting 1-, and 2-year OS in patients with SRCC in the training **(B)** and validation sets **(F)**. Validation of the OS **(C, G)** nomograms using receiver operating characteristic (ROC) curves. The 1- and 2-year OS decision curve analysis (DCA) curve of nomogram, and TNM stage in patients with SRCC in the training **(D, E)** and validation sets **(H, I)**. **p < 0.01; ***p< 0.001.

**Figure 5:**
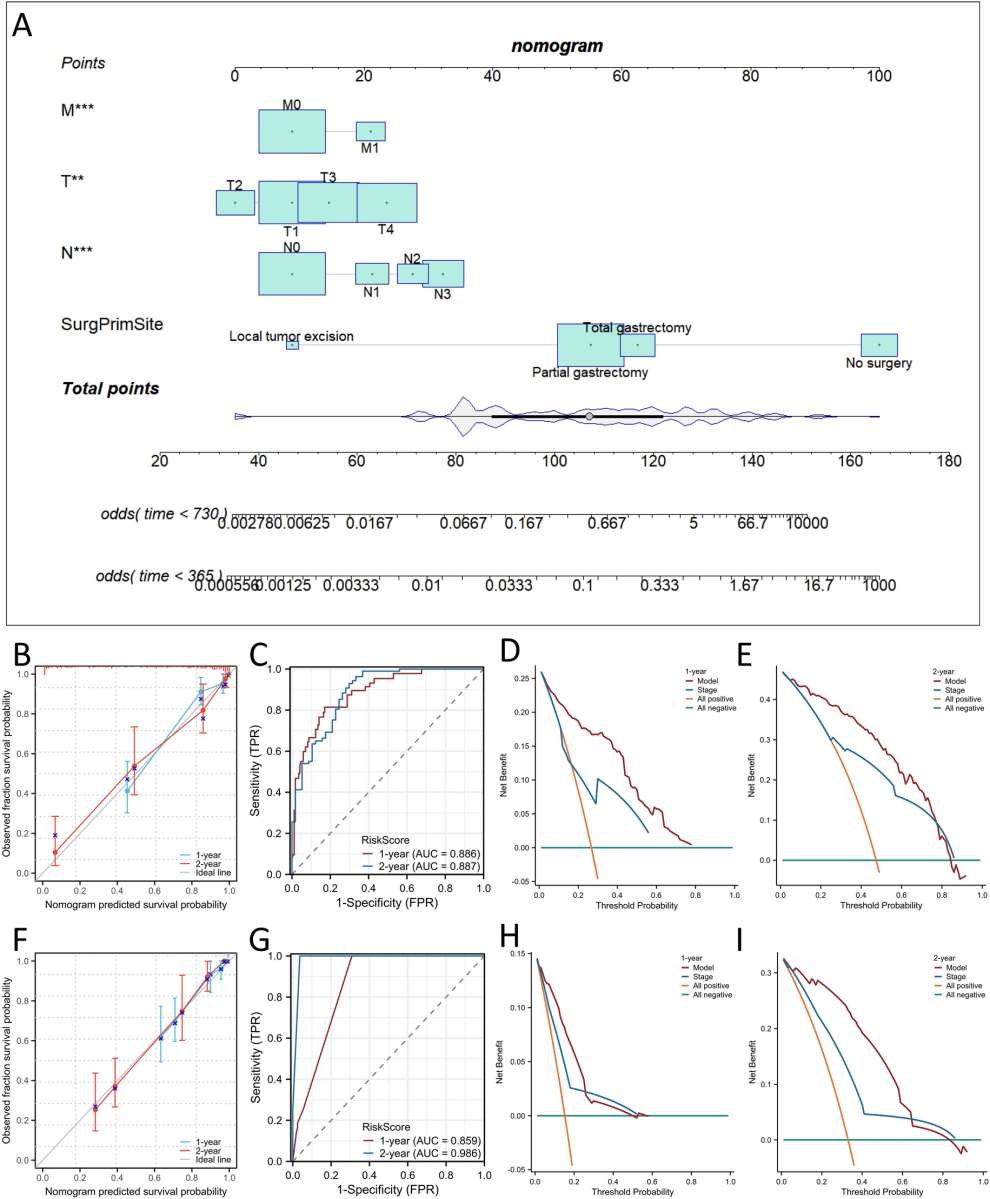
Construction of nomograms for predicting Cancer Specific Survival (CSS) in SRCC patients and validation of their efficacy and accuracy. Nomograms predicting 1- and 2-year CSS **(A)** in patients with SRCC. Calibration plot of the nomogram for predicting 1-, and 2-year CSS in patients with SRCC in the training **(B)** and validation sets **(F)**. Validation of the CSS **(C, G)** nomograms using receiver operating characteristic (ROC) curves. The 1- and 2-year CSS decision curve analysis (DCA) curve of nomogram, and TNM stage in patients with SRCC in the training **(D, E)** and validation sets **(H, I)**. **p < 0.01; ***p < 0.001.

Considering the results of the multivariate Cox regression analysis above, all the significant factors were used to construct a nomogram to predict the probability of 1- and 2-year OS and CSS in patients with SRCC based on the sum of all the scores, as shown in **Figures 4A** and **5A**. The nomogram showed that the surgical approach had the most significant impact, while M stage had the most negligible impact on OS and CSS. ROC curve analysis of the nomogram revealed that the nomogram had a high discriminative ability for predicting the probability of benefit, with AUC values for 1- and 2-year OS in the training set of 0.897 and 0.878, and AUC values for the 1-year and 2-year OS in the external validation set were 0.863 and 0.950, respectively (**Figures 4C, G**). Calibration curves can be used to evaluate the accuracy of the model’s predicted probability compared with the actual probability. In this study, the calibration curve closely followed the reference line, which indicates relatively good consistency between the predicted and actual results (**Figures 4B, F**). DCA is often used to evaluate practical clinical decisions, and thus we performed DCA to compare the clinical benefits of the nomogram and the AJCC staging system, and we found that the nomogram has clinical value for SRCC patients (**Figures 4D, E**). Similarly, the AUC values for 1- and 2-year CSS in the training set were 0.886 and 0.887, and the AUC values for the 1-year and 2-year CSS in the external validation set were 0.59 and 0.986, respectively (**Figures 5C, G**). Calibration curves revealed that the survival rate predicted by the nomogram was consistent with the actual observed results (**Figures 5B, F**). Furthermore, the DAC curves demonstrated that the two nomograms had good net clinical benefits (**Figures 4D, E, H, I**, **5D, E, H, I**).

We also comprehensively compared the nomogram used to predict OS and CSS with the AJCC staging system. For predicting the OS rate, the C-index of the nomogram was 0.810 in the training set (95% CI, 0.802−0.876), 0.840 in the bootstrap validation set (95% CI, 0.800−0.873), and 0.859 in external validation set (95% CI, 0.802-0.916), and thus the nomogram showed greater statistical power than AJCC TNM staging [0.775 (95% CI, 0.703−0.810), *p <*0.001; 0.778 (95% CI, 0.706−0.816), *p <*0.001; 0.796(95% CI,(0.711-0.881), *p <*0.001]. For CSS prediction, the C-indices of the nomograms in the training set, bootstrap validation set and the external validation set were 0.823 (95% CI, 0.801−0.879),0.843 (95% CI, 0.798−0.877) and 0.868 (95% CI, 0.811−0.925), respectively, which were greater than those of the AJCC TNM staging system [0.728 (95% CI, 0.698−0.811), *p <*0.001; 0.724 (95% CI, 0.699−0.811), *p <*0.001; 0.803 (95% CI, 0.781−0.825), *p* <0.001]. In addition, our nomogram produced minimum AIC values compared with those of the AJCC staging system (**Supplementary Table 2**). The results showed that our nomogram had a robust and more accurate prediction ability than the traditional AJCC staging system.

### Subgroup analysis stratified by SRCC patient risk

**Figure 6:**
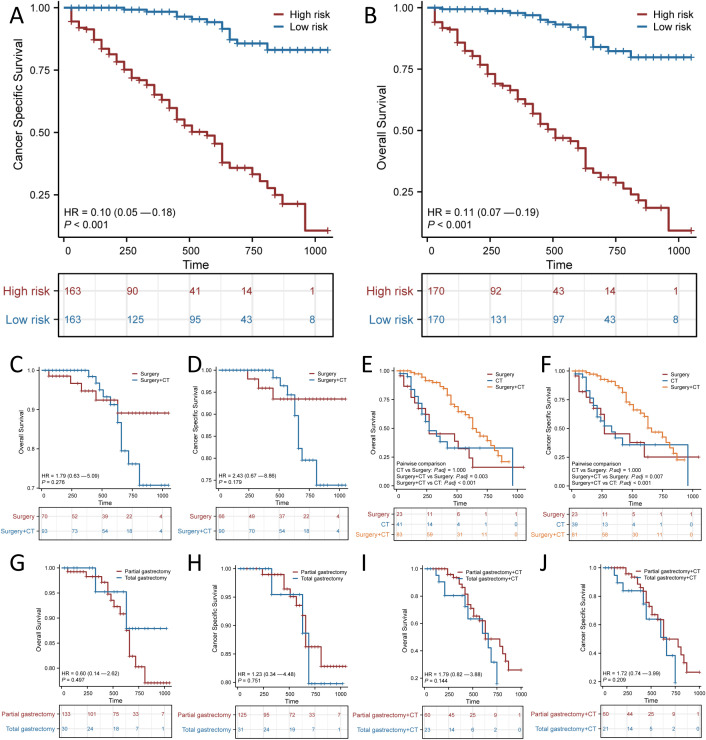
Kaplan–Meier curves for predicting Overall survival (OS) and Cancer Specific Survival (CSS) based on the new risk stratification system. Kaplan–Meier analysis of the OS **(A)** and CSS **(B)** of the high and low score groups. OS **(C, E)** and CSS **(D, F)** of different treatment modalities for patients in the low-risk and high-risk groups. OS **(G)** and CSS **(H)** of different surgical modalities for low-risk patients. OS **(I)** and CSS **(J)** of different surgical modalities for high-risk patients receiving chemotherapy (CT).

SRCC patients were classified into high-risk and low-risk groups according to the median scores of the nomogram for predicting prognosis. KM curves revealed a statistically significant difference in both OS and CSS between the two subgroups (*p* < 0.001 for OS; *p* < 0.001 for CSS; **Figures 6A, B**). In addition, both surgery and chemotherapy are important treatments for patients with SRCC. We used KM curves to clarify the effect of treatments, including surgery and chemotherapy, in the two risk-stratified subgroups mentioned above. Neither OS nor CSS was improved by surgery combined with chemotherapy compared with surgery alone in the low-risk group (**Figures 6C, D**), and surgery combined with chemotherapy was superior to surgery alone or chemotherapy alone in the high-risk group (**Figures 6E, F**). This finding indicated that surgery alone is sufficient for the low-risk group and that surgery combined with chemotherapy may be beneficial for the high-risk group. We further clarified the effect of surgical approach on different subgroups of patients with SRCC and found that while surgery played an important role in the low- and high-risk groups, subjectively enlarged total gastrectomy did not improve OS or CSS (**Figures 6G–J**).

## Discussion

In recent years, despite a decrease in the incidence of gastric cancer, the incidence of SRCC has increased (31). Increasing numbers of studies in Asian countries have demonstrated that the prognosis of SRCC is dependent on pathological grade and stage. These studies have shown that SRCC has a more favorable prognosis than non-SRCC in the early stages, whereas non-SRCC has a more favorable prognosis in the late stages (32–35). Nevertheless, some studies have failed to identify any significant differences between these two types of cancer (36). Regarding the diametrically opposed survival trends of SRCC in the early and late stages, some researchers have postulated that the early stage of SRCC is typified by a latent state with low invasiveness but that when tumor cells invade the muscularis propria, the tumor’s invasiveness increases and accelerates markedly, thereby increasing the risk of peritoneal metastasis (37, 38). Particularly, reduced expression of E-cadherin, which is encoded by the CDH1 gene, is associated with a lack of cell-to-cell adhesion and an elevated risk of metastasis (39–44). Given the distinct biological behaviors of SRCC in comparison to AC, and its limited clinical benefit in relation to conventional chemotherapy and targeted therapy, screening the population for potential immune benefit based on SRCC single-cell sequencing or genetic testing represents an effective strategy for harnessing the potential of immunotherapy (45, 46).

In all, 559 patients with SRCC from the SEER database were included in our study. More patients with SRCC had T4 stage, N2 stage, and stage III and IV disease, which is consistent with previous studies that have reported that SRCC is commonly diagnosed at an advanced stage. Our findings indicate that the clinical features of SRCC are distinct from those of AC. One notable difference is that, compared with AC, the age of onset of SRCC is significantly higher in individuals younger than 45 years. Additionally, a discrepancy was also noted in the gender distribution between the two cancer types. AC is typically regarded as a male-dominated cancer and accounts for approximately two-thirds of all gastric cancer cases, whereas in SRCC, approximately half of the patients are women. Previous research has indicated that younger women exhibit higher levels of estrogen receptors, which suggests that SRCC has a greater affinity for estrogen. Therefore, sex hormones are postulated to contribute to age and gender differences (47, 48).

The present study revealed that the median OS and median CSS rates of patients with SRCC were 22 months and 24 months, respectively. These rates are considerably shorter than those observed in patients diagnosed with AC, and these findings are consistent with those of previous studies. A noteworthy observation emerged from the analysis of the data on SRCC and AC following PSM. Despite the significant difference in the CSS between the two groups, the OS was not significantly different, although a difference of 11 months was observed in the median OS between the two cancers. This suggests that the shorter OS of SRCC patients before PSM may be attributed to inconsistencies in the baseline data. The relatively advanced stage of SRCC patients according to the pre-PSM baseline data may explain the poorer prognosis of these patients. To identify further potential prognostic factors, univariate and multivariate analyses were conducted, which revealed that T4 stage, N2-3 stage, metastasis, and no surgery were independent risk factors for SRCC.

Although SRCC is a highly aggressive disease, data suggest that early SRCC may be no more aggressive than early non-SRCC. Consequently, surgery remains the mainstay of treatment. Studies have shown that patients who undergo radical surgery have a better prognosis than those who undergo palliative surgery or no surgery (49, 50). Although this was an observational study, after using PSM to reduce bias, we found that surgical treatment improved OS and CSS in patients with SRCC. This suggests that surgery is the most effective treatment, at least for patients with early-stage SRCC without metastases. In addition, T stage and tumor size have been found to be independent predictors of lymph node metastasis in early SRCC, and as such, enzyme secretion detection (ESD) resection may be indicated for T1a tumors, as this approach can preserve organ function and is associated with a lower rate of cancer recurrence (22, 51, 52). Nevertheless, in patients with T1b tumors, the characteristics of signet ring cell carcinoma result in a high rate of lymph node spread, with a lymph node positivity rate exceeding 10%, even in tumors smaller than 1.0 cm. In such cases, surgery is a more thorough and advantageous treatment.

Landmark clinical trials on adjuvant treatment of gastric cancer (INT-0116 and MAGIC) revealed that chemotherapy (epirubicin, cisplatin and fluorouracil) in conjunction with surgery was associated with a superior prognosis compared with surgery alone (53–57). The efficacy of radiotherapy remains a matter of contention; the results of the ARTIST study indicated no discernible enhancement in the 5-year OS rate of gastric cancer (GC) patients undergoing adjuvant capecitabine/cisplatin chemotherapy in conjunction with radiotherapy following radical D2 surgery (58–62). A review of survival trends in patients with SRCC over the past decade has indicated an improvement in the 2-year relative survival. This improvement in survival may be attributed to the recent development of perioperative chemotherapy. A recent study demonstrated that patients with advanced SRCC treated with TEFOX chemotherapy had an OS rate of 65%, with a median survival of 14 months (63, 64). In contrast to other similar studies, our investigation concentrated on chemotherapy and surgical treatment, which are integral components of systemic therapy and are therefore more representative of medical reality. This study demonstrated that chemotherapy was not an independent protective factor. However, the combination of surgery and chemotherapy was found to be more efficacious than either treatment alone in patients at high risk and had significant prognostic implications.

The nomogram, which integrates multiple parameters to assess survival probability, is more precise than the current AJCC staging system and is therefore regarded as an alternative and even a novel staging system. Previous studies have demonstrated the prognostic value of nomograms in SRCC; however, those studies either did not incorporate treatment data or they failed to fully utilize the nomogram to assess risk and inform the choice of treatment (16, 28, 29, 65). In this study, we constructed a nomogram for predicting OS and CSS in SRCC patients based on T stage, N stage, M stage, and surgical modality. We then transformed the Cox regression results into visual graphs, which increased readability of the prediction model and facilitated the assessment of OS and CSS in SRCC patients. In recent studies, several SRCC prognostic nomograms have been developed (16, 52). In comparison, the nomogram developed in this study demonstrates superior prognostic prediction performance in both internal and external validations. Furthermore, the nomogram demonstrated greater accuracy in both the C-index and AIC than did the AJCC TNM staging system. Our nomogram also enables the identification of patients with varying risk profiles, facilitating the implementation of tailored treatment approaches for this tumor type, the incidence of which continues to increase. Considering these points, patients who might not benefit from treatment should be identified. Therefore, our study classified the benefits in terms of OS according to a nomogram and explored personalized treatment strategies for SRCC patients in different risk stratification groups from the perspective of prognostic benefit. In accordance with our findings based on the new risk stratification system, it is evident that in the high-risk group, patients who underwent surgery in combination with chemotherapy exhibited a significantly higher survival rate than those who were treated with either surgery alone or chemotherapy alone. However, for the low-risk group, no discernible difference was observed in survival outcomes between those who received combination chemotherapy and those who underwent surgery. Therefore, patients with SRCC in the low-risk group may undergo surgery alone, without the necessity of chemotherapy, whereas chemotherapy plus surgery might be a better approach for patients with SRCC in the high-risk group.

SRCC is malignant and prone to submucosal infiltration and lymph node metastasis, and clinical surgeons believe that total gastrectomy can improve patient prognosis by increasing the extent of resection. The results of our study indicated that total gastrectomy did not result in superior outcomes relative to partial gastrectomy in either the low- or high-risk groups. Intraoperative pathological examination of the surgical margins was found to be an effective method for reducing residual tumor and short-term recurrence. To avoid incomplete clearance of positive lymph nodes, membrane dissection-guided laparoscopic 4d, 5, 6, and 12a lymph node clearance was found to be a safe and feasible approach. Furthermore, proximal gastrectomy confers several advantages in terms of weight maintenance, postoperative anemia and nutrition (including vitamin B12, protein, albumin and cholesterol) (66).

Notably, this study is subject to several unavoidable limitations. First, the limited data in the SEER database to collect treatment-specific information such as the strategy, sequence, and dose of chemotherapy, and extent of radical lymphadenectomy dissection, which may limit the value of that can be derived from this comprehensive risk adjustment methodologies. In addition, the database also these factors may have a significant impact on patient prognosis. Second, the PSM analysis used in this study has limitations. Propensity scores do not easily satisfy equilibrium if the sample is small. Furthermore, it does not effectively deal with selectivity bias caused by unobservable or unobservable factors. Third, retrospective analyses are inevitably susceptible to selection bias and competing risks. Attempts have been made to minimize the effects of confounding factors through the use of statistical methods such as PSM analyses, multivariate analyses, and subgroup analyses. While these techniques are designed to control for bias, they are still unlikely to eliminate all errors. The establishment of a global SRCC database will allow for more comprehensive data and the development of appropriate treatment strategies.

## Conclusion

In conclusion, we compared the differences in basic features and prognosis between patients with SRCC and those with AC and constructed and validated a nomogram of OS and CSS for SRCC, which was more accurate than the TNM staging system. Furthermore, a subgroup analysis according to risk stratification based on the nomogram can help clinicians personalize treatment for different subgroups of SRCC patients. The results of this study highlight the necessity for prospective clinical trials to clarify the appropriate treatment for this gastric cancer subtype. It is recommended that retrospective and prospective studies be conducted using the Global SRCC Database to obtain more comprehensive data and to develop appropriate treatment strategies.

## Data Availability

The original contributions presented in the study are included in the article/**Supplementary Material**. Further inquiries can be directed to the corresponding authors.

## Supplementary material

Supplementary Figure 1 Propensity score distribution between patients with signet ring cell carcinoma (SRCC) and adenocarcinoma (AC) before and after propensity score matching. **(A)** histogram and **(B)** line graph.

Supplementary Figure 2 Kaplan-Meier survival curves of patients with adenocarcinoma (AC) and signet ring cell carcinoma (SRCC) at different pathological stages after propensity score matching. Overall survival (OS) **(A)** and Cancer Specific Survival (CSS) **(E)** in stage I patients; OS **(B)** and CSS **(F)** in stage II patients; OS **(C)** and CSS **(G)** in stage III patients; and OS **(D)** and CSS **(H)** in stage IV patients.
