# Supplementary figures and images for: Prognostic prediction and treatment options for gastric signet ring cell carcinoma: a SEER database analysis
Source: Front Oncol. 2024 Oct 21;14:1473798. doi: 10.3389/fonc.2024.1473798 (PMC11532132; doi:10.3389/fonc.2024.1473798)

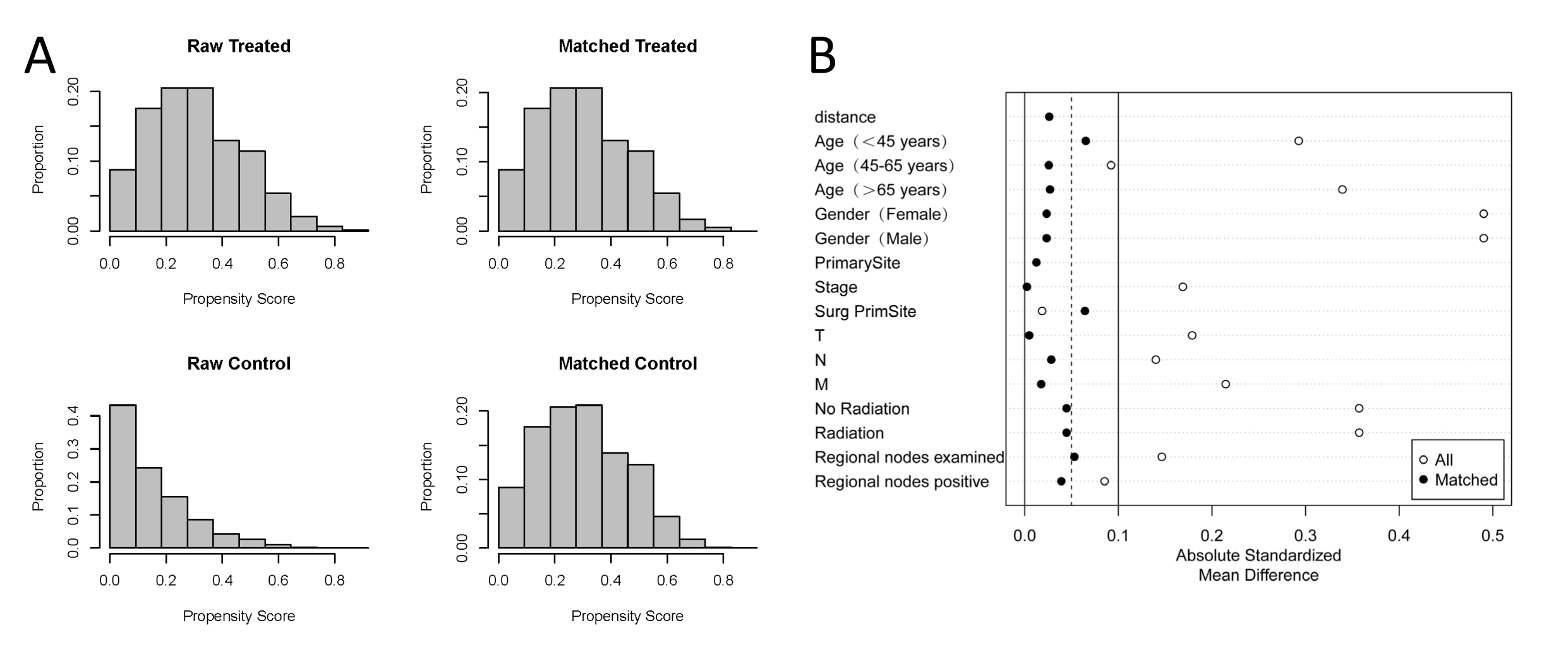

Supplement: Supplementary Figure 1 — Propensity score distribution between patients with signet ring cell carcinoma (SRCC) and adenocarcinoma (AC) before and after propensity score matching. (A) histogram and (B) line graph. [file Image1.tif]

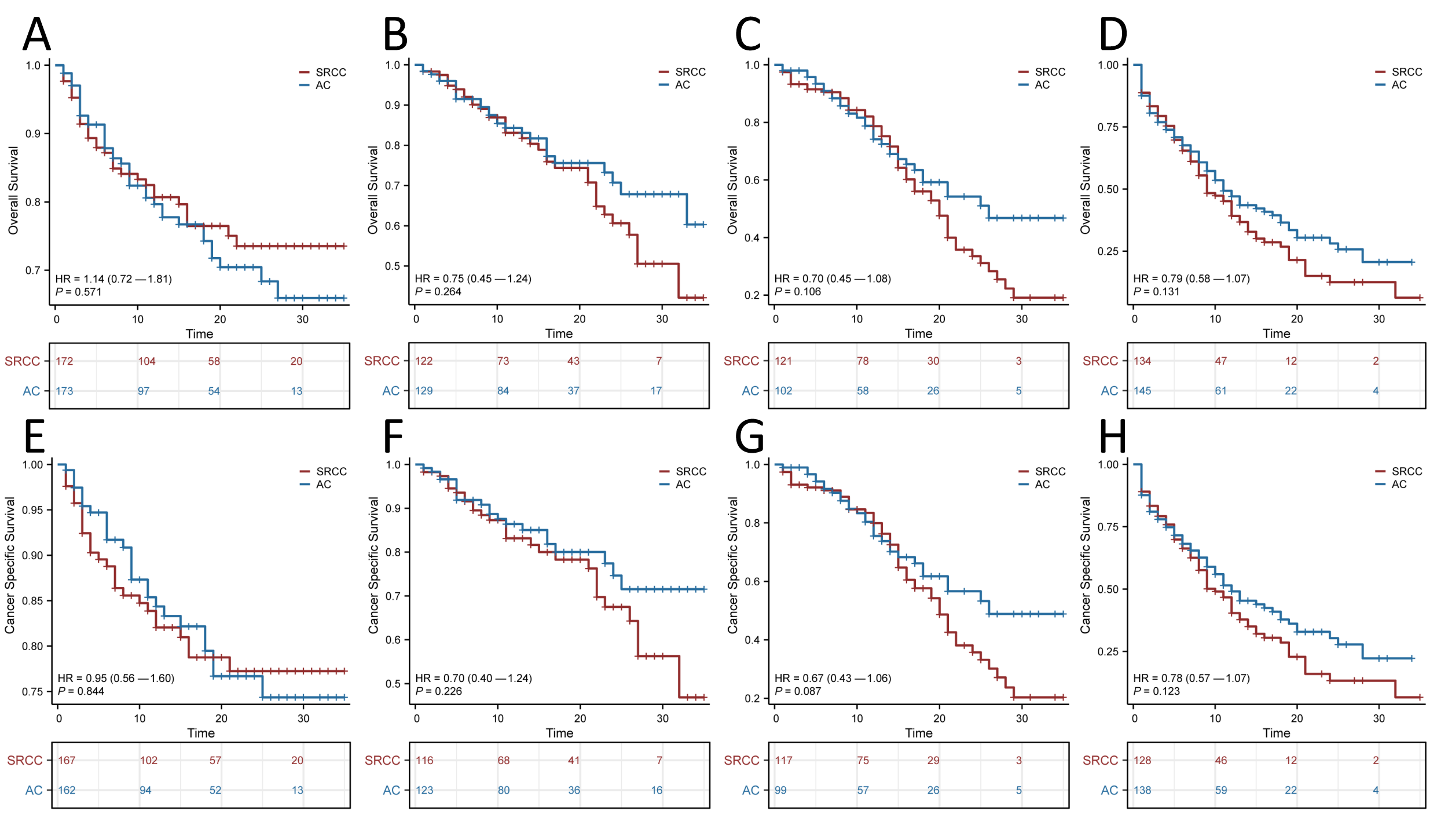

Supplement: Supplementary Figure 2 — Kaplan-Meier survival curves of patients with adenocarcinoma (AC) and signet ring cell carcinoma (SRCC) at different pathological stages after propensity score matching. Overall survival (OS) (A) and Cancer Specific Survival (CSS) (E) in stage I patients; OS (B) and CSS (F) in stage II patients; OS (C) and CSS (G) in stage III patients; and OS (D) and CSS (H) in stage IV patients. [file Image2.tif]
