# Supplementary material for: Prognostic prediction and treatment options for gastric signet ring cell carcinoma: a SEER database analysis
Source: Front Oncol. 2024 Oct 21;14:1473798. doi: 10.3389/fonc.2024.1473798 (PMC11532132; doi:10.3389/fonc.2024.1473798)
Supplement: Supplementary file 3 [file Table1.docx]

**Supplementary Table 1 Univariate and multivariate analyses of the SRCC patients for cancer-specific survival**

| Characteristics | Total(N) | Univariate analysis | |  | Multivariate analysis | |
| --- | --- | --- | --- | --- | --- | --- |
|  |  | Hazard ratio (95% CI) | *P*- value |  | Hazard ratio (95% CI) | *P*- value |
| Age | 326 |  |  |  |  |  |
| <45 years | 48 | Reference |  |  |  |  |
| 45-65 years | 128 | 1.527 (0.780 - 2.989) | 0.217 |  |  |  |
| >65 years | 150 | 1.310 (0.667 - 2.575) | 0.433 |  |  |  |
| Race | 326 |  |  |  |  |  |
| Other (American Indian/AK Native, Asian/Pacific Islander) | 104 | Reference |  |  |  |  |
| White | 196 | 0.963 (0.609 - 1.522) | 0.871 |  |  |  |
| Black | 24 | 1.083 (0.418 - 2.811) | 0.869 |  |  |  |
| Unknown | 2 | 1.950 (0.264 - 14.392) | 0.513 |  |  |  |
| Gender | 326 |  |  |  |  |  |
| Female | 183 | Reference |  |  | Reference |  |
| Male | 143 | 1.594 (1.044 - 2.435) | **0.031** |  | 1.421 (0.902 - 2.239) | 0.130 |
| Primary Site | 326 |  |  |  |  |  |
| Cardia | 23 | Reference |  |  |  |  |
| Fundus of stomach | 10 | 0.000 (0.000 - Inf) | 0.995 |  |  |  |
| Body of stomach | 55 | 0.588 (0.243 - 1.423) | 0.239 |  |  |  |
| Gastric antrum | 103 | 0.736 (0.337 - 1.609) | 0.443 |  |  |  |
| Pylorus | 21 | 0.397 (0.119 - 1.323) | 0.133 |  |  |  |
| Lesser curvature of stomach | 37 | 0.456 (0.171 - 1.218) | 0.117 |  |  |  |
| Greater curvature of stomach | 9 | 1.192 (0.357 - 3.973) | 0.775 |  |  |  |
| Overlapping lesion of stomach | 28 | 0.698 (0.262 - 1.863) | 0.474 |  |  |  |
| Stomach | 40 | 0.734 (0.299 - 1.801) | 0.500 |  |  |  |
| Surg Prim Site | 326 |  |  |  |  |  |
| Partial gastrectomy | 205 | Reference |  |  | Reference |  |
| No surgery | 59 | 6.611 (4.092 - 10.681) | **< 0.001** |  | 25.811 (10.704 - 62.237) | **< 0.001** |
| Local tumor excision | 7 | 0.000 (0.000 - Inf) | 0.994 |  | 0.000 (0.000 - Inf) | 0.995 |
| Total gastrectomy | 55 | 1.369 (0.746 - 2.513) | 0.311 |  | 1.672 (0.902 - 3.099) | 0.103 |
| T | 326 |  |  |  |  |  |
| T1 | 109 | Reference |  |  | Reference |  |
| T2 | 35 | 0.641 (0.187 - 2.204) | 0.481 |  | 0.524 (0.147 - 1.866) | 0.318 |
| T3 | 93 | 2.577 (1.408 - 4.718) | **0.002** |  | 1.541 (0.796 - 2.981) | 0.199 |
| T4 | 89 | 3.885 (2.152 - 7.013) | **< 0.001** |  | 3.031 (1.452 - 6.327) | **0.003** |
| N | 326 |  |  |  |  |  |
| N0 | 177 | Reference |  |  | Reference |  |
| N1 | 44 | 0.714 (0.319 - 1.598) | 0.412 |  | 2.465 (0.864 - 7.031) | 0.092 |
| N2 | 38 | 1.636 (0.899 - 2.976) | 0.107 |  | 3.802 (1.464 - 9.877) | **0.006** |
| N3 | 67 | 2.178 (1.325 - 3.582) | **0.002** |  | 5.483 (2.250 - 13.359) | **< 0.001** |
| M | 326 |  |  |  |  |  |
| M0 | 275 | Reference |  |  | Reference |  |
| M1 | 51 | 6.327 (3.932 - 10.180) | **< 0.001** |  | 2.104 (1.220 - 3.627) | **0.007** |
| Surg/Rad Seq | 326 |  |  |  |  |  |
| No | 294 | Reference |  |  |  |  |
| Yes | 32 | 1.308 (0.694 - 2.464) | 0.406 |  |  |  |
| Chemotherapy | 326 |  |  |  |  |  |
| No/Unknown | 116 | Reference |  |  |  |  |
| Yes | 210 | 1.105 (0.706 - 1.730) | 0.662 |  |  |  |
| Tumor Size Summary | 326 | 1.004 (1.001 - 1.007) | **0.006** |  | 1.000 (0.995 - 1.005) | 0.895 |

Abbreviations: SRCC, Signet ring cell carcinoma; T, tumor; N, node; M, metastasis
