# Supplementary material for: Prognostic prediction and treatment options for gastric signet ring cell carcinoma: a SEER database analysis
Source: Front Oncol. 2024 Oct 21;14:1473798. doi: 10.3389/fonc.2024.1473798 (PMC11532132; doi:10.3389/fonc.2024.1473798)
Supplement: Supplementary file 4 [file Table2.docx]

**Supplementary Table 2 The AIC, AUC, and C‐index of the nomograms and TNM Stage system in OS and CSS prediction for SRCC patients**

**OS:**

| **Items** | **Nomogram** | **TNM system** | ***P*-value** |
| --- | --- | --- | --- |
| Training set |  |  |  |
| AIC | 917.362 | 977.372 |  |
| C-index (95% CI) | 0.810 (0.802-0.876) | 0.775 (0.703-0.810) | <0.001 |
| AUC | 0.897 (0.852-0.941) | 0.794 (0.722-0.866) | <0.001 |
| Bootstrap- Validation Set |  |  |  |
| AIC | 918.967 | 979.996 |  |
| C-index (95% CI) | 0.840 (0.800-0.874) | 0.778 (0.706-0.816) | <0.001 |
| AUC | 0.807(0.759-0.850) | 0.727 (0.670-0.794) | <0.001 |
| External validation set |  |  |  |
| AIC | 920.382 | 982.341 |  |
| C-index (95% CI) | 0.859 (0.802-0.916) | 0.796 (0.711-0.881) | <0.001 |
| AUC | 0.903 (0.829-0.977) | 0.768 (0.689-0.847) | <0.001 |

Abbreviations: SRCC, Signet ring cell carcinoma; TNM, Tumor-Node-Metastasis; AIC, Akaike information criterion; C-index, concordance index; AUC, Area Under the Curve; OS, Overall survival; CSS: Cancer Specific Survival

**CSS:**

| **Items** | **Nomogram** | **TNM system** | ***P*-value** |
| --- | --- | --- | --- |
| Training set |  |  |  |
| AIC | 775.986 | 976.851 |  |
| C-index (95% CI) | 0.823 (0.801-0.879) | 0.728 (0.698-0.811) | <0.001 |
| AUC | 0.886 (0.835-0.937) | 0.803 (0.727-0.879) | <0.001 |
| Bootstrap- Validation Set |  |  |  |
| AIC | 776.009 | 978.258 |  |
| C-index (95% CI) | 0.842 (0.798-0.8765) | 0.724 (0.699-0.811) | <0.001 |
| AUC | 0.806 (0.766-0.858) | 0.716 (0.655-0.797) | <0.001 |
| External validation set |  |  |  |
| AIC | 811.452 | 874.402 |  |
| C-index (95% CI) | 0.868 (0.811-0.925) | 0.803 (0.781-0.825) | <0.001 |
| AUC | 0.859 (0.829-0.889) | 0.843 (0.803-0.883) | <0.001 |

Abbreviations: SRCC, Signet ring cell carcinoma; TNM, Tumor-Node-Metastasis; AIC, Akaike information criterion; C-index, concordance index; AUC, Area Under the Curve; OS, Overall survival; CSS: Cancer Specific Survival
